# A Governance Framework to Assist with the Adoption of Sensing Technologies in Construction

**DOI:** 10.3390/s22010260

**Published:** 2021-12-30

**Authors:** Mona Arabshahi, Di Wang, Yufei Wang, Payam Rahnamayiezekavat, Weichen Tang, Xiangyu Wang

**Affiliations:** 1 School of Design and Built Environment, Curtin University, Perth, WA 6102, Australia; Mona.Arabshahi@curtin.edu.au (M.A.); 20760096@student.curtin.edu.au (Y.W.); 2 Institute for Smart City of Chongqing University in Liyang, Chongqing University, Liyang 213300, China; 202116131002@cqu.edu.cn; 3 School of Built Environment, Western Sydney University, Penrith, NSW 2751, Australia; p.zekavat@westernsydney.edu.au; 4 School of Civil Engineering, Chongqing University, Chongqing 400045, China; 202116131275@cqu.edu.cn

**Keywords:** sensing technologies, governance framework, online survey, semi-structured interviews, Partial Least Squares Structural Equation Modelling, triangulation analysis

## Abstract

Sensing technologies present great improvements in construction performance including the safety, productivity, and quality. However, the corresponding applications in real projects are far behind compared with the academically research. This research aims to discover dominate influence factors in the sensing technologies adoption and ultimately develop a governance framework facilitating adoption processes. The framework is dedicated on general sensing technologies rather than single sensor in previous framework studies. To begin with, the influence factors of sensing technologies and other similar emerging technologies are summarised through a review. Then, a mixed methods design was employed to collect quantitative data through an online survey, and qualitative data through semi-structured interviews. Findings of the quantitative method reveal that the most widely implemented sensing technologies are GPS and visual sensing technology, but they’re still not adopted by all construction companies. Partial Least Squares Structural Equation Modelling reveals that supplier characteristics have the highest effect in all influence factors. Qualitative method was adopted to investigate perceptions of construction stakeholders on the major decision-making considerations in the adoption process. Ultimately, a triangulation analysis of findings from the literature review, online survey and interviews resulted in the governance framework development. The overarching contribution of this research focus on the general adoption of sensing technologies rather than the adoption of a specific sensor. Therefore, the governance framework can assist with the decision-making process of any sensing technology adoption in construction.

## 1. Introduction

The construction industry is regarded as an information-dependent and information-intensive domain because of the complexity and dynamic nature of construction projects [1]. The conventional data collection process is labour-intensive, costly, and error-prone, which can’t meet the increasing requirements of modern construction management [2,3]. A sensor is a device converting input from a physical condition into an electronic signal. The sensing technology has been employed in automated data acquisition, which revolutionizes data collection, transmission, and analysis in the construction industry. It is promising to be used accompanied by the development of advanced construction technology such as 3D printing technology [4,5,6,7]. Sensing technologies are also employed in the experimental investigation on rehabilitation of corroded reinforced concrete columns [8,9,10]. For structure built with new cement materials such as solid wastes incorporated concrete, the structural monitoring is rather important [11,12,13,14,15]. By combining with artificial intelligence, the data derived from sensors can be efficiently and effectively analysed to guide the design the construction [16,17,18,19,20]. In general, advanced sensor technology has been a focus of research owing to great potentials in human errors reduction, construction project management, and construction performance enhancement [21,22]. However, the corresponding applications are far behind in real projects.

Sensing technology adoption has been challenged by the fragmented and temporary nature of construction projects, along with technology-related, adoption process-related, and human-related factors [23,24]. The real-time locating systems (RTLS) have been proved to be valid in construction productivity enhancement. However, the implementation is slow because key factors have been overlooked, such as cost and deployment [25,26,27]. Besides, laser scanners are effective in construction activities monitoring by laser signals from a rotating laser photon source [28,29]. However, the deployment is restricted by factors containing a clear line of sight, long data processing time, and high data storage capacity. In general, sensing technology adoption is hampered by operating costs, lack of well-trained staff, and technology immaturity [30,31].

Inductive reasoning will guide this research from the perceptions of construction stakeholders (observation) to the suitability assessment of sensing technologies (theory) presented in the framework. Mixed qualitative-quantitative research methods have been used in identifying construction stakeholders’ attitudes towards the new technologies and associated decision making processes [32]. Besides, Hong et al. [33] has employed the mixed qualitative-quantitative research methods for exploring barriers to BIM adoption. Therefore, a survey on dominant factors influencing the adoption and key factors considered during the adoption process is in great demand.

**Figure 1:**
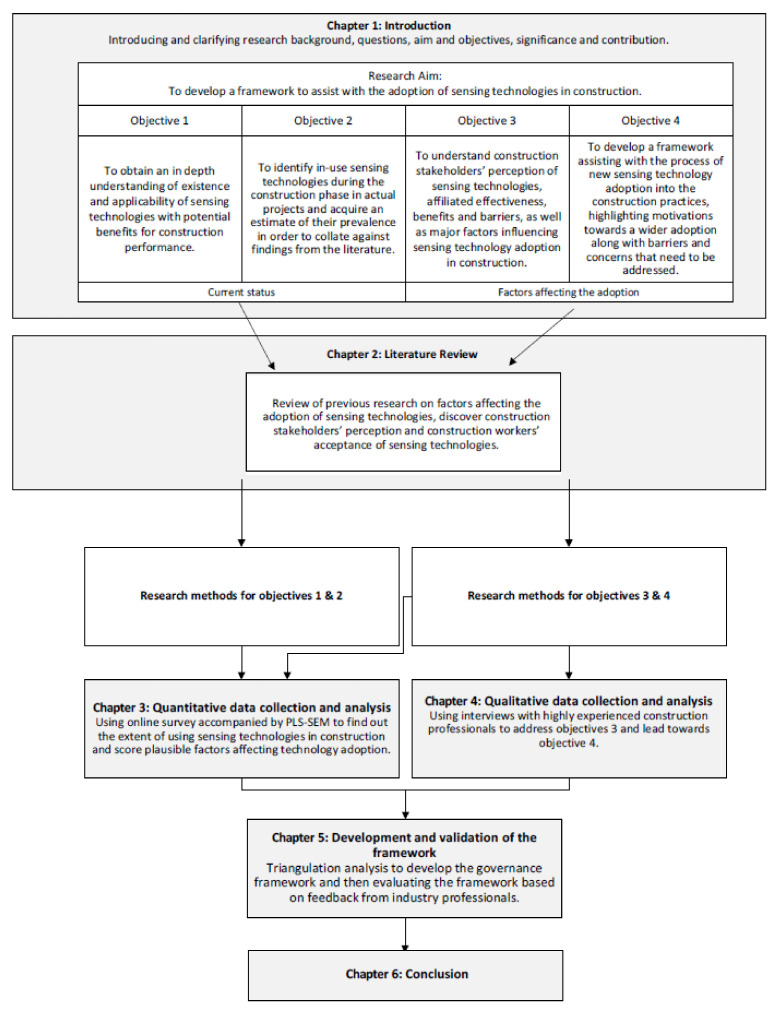
Structure of the thesis.

This research is designed to find out the current status of sensing technologies and facilitate the adoption process in the construction industry. Odubiyi et al. [34] indicated that a wide range of challenges in advanced technologies application is related to people within the construction industry. Construction stakeholders experienced in sensing technologies and involved in the adoption process are ideal subjects. The flow of the six chapters of this thesis is depicted in Figure 1. To begin with, a literature review is conducted with 187 potential articles on types of sensing technologies and their applications in construction. Then, an online survey articulates the current status of sensing technologies in real projects. Next, interviews of the perceptions from construction stakeholders will support and supplement the findings of the online survey. Besides, analysis of the interviews will identify the major decision-making considerations during the sensing technology adoption process. A governance framework is envisaged to highlight the benefits from sensing technologies, foresee the risks during the application, and facilitate decision-making about the adoption. Lastly, the governance framework will be validated and supplemented by industry feedback from construction professionals. The governance framework will prepare a pathway for wider adoption of sensing technologies, which will improve the construction industry on safety, quality, and efficiency.

## 2. Literature Review

**Figure 2:**
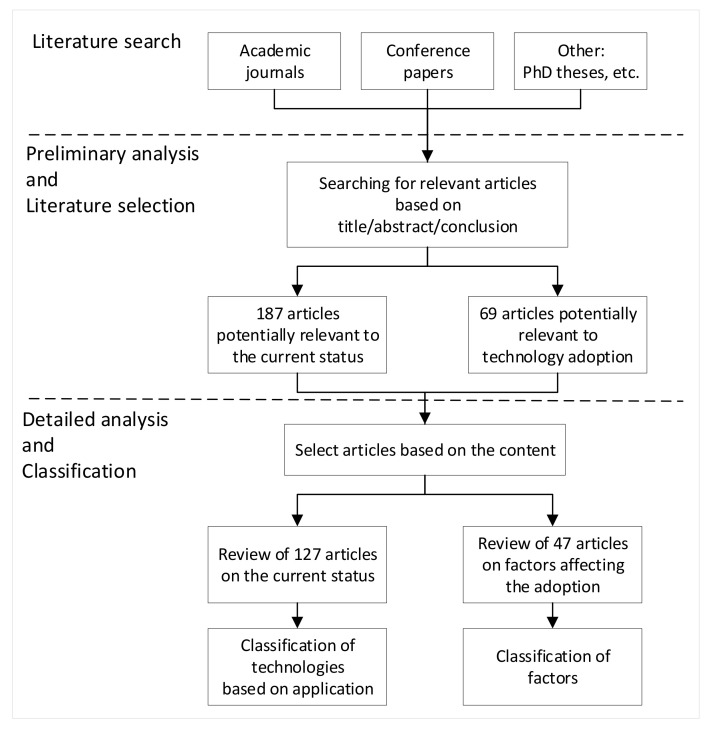
Method for literature review.

Sensing technologies have been academically researched but their adoption is slow in construction projects [35]. Inadequate understanding and neglect of innovative sensing technologies restrict real project adoption despite great benefits in construction performance improvement [36,37]. Besides, information about the deployment, time, cost, and accuracy of in-use sensing technologies such as real-time locating systems is insufficient [38]. The literature review has investigated the current status of common sensing technologies and explored the factors influencing their adoption. Selected sensing technologies contained Global Positioning System, Radio Frequency Identification, Ultra-wideband, Fiber Optic Sensing, pressure sensing, temperature sensing, visual sensing, and three-dimensional scanning technology. Of 187 potential articles on types of sensing technologies and their applications in construction, 127 were selected to classify technologies based on their applications. Of 69 articles relevant to the adoption of technology, 47 were subsequently analysed to identify factors affecting the adoption of sensing technologies in construction. The process followed for the literature review in this chapter was shown in Figure 2. Lastly, factors influencing the sensing technology adoption are concluded in Table 1.

## 3. Quantitative Data Collection and Analysis

Surveys are powerful and common means of quantitative data collection to understand stakeholders’ views of various aspects of construction management. For example, Jacobs et al. [68] have perceived construction employees’ willingness to use wearable sensors through a survey. Furthermore, Liu et al. [69] have developed a BIM-aided construction waste minimisation framework based on data collected from questionnaire surveys and follow up interviews. Therefore, a survey is reasonable in this research.

Quantitative data collected from an online survey included the current status of sensing technologies and factors affecting their adoption. The key eligibility to participate in the research was the considerable experience in construction and experience in using sensing technologies. A response rate of 31.5% was achieved with 82 complete questionnaires from 261 samples was acceptable in construction studies [70]. Among 82 survey respondents, 30 (37%) were from the building sector, 34 (41%) from the infrastructure sector, and 18 (22%) from the industrial construction sector. Factors significantly affecting sensing technologies adoption will be extracted from Table 1. The descriptive analysis of the current status of sensing technologies confirmed the slow adoption in literature.

### 3.1. Current Status of Sensing Technologies Implementation

#### 3.1.1. Global Positioning System (GPS)

GPS is recognized as one of the most frequently used sensing technologies in all three sectors of construction: building construction, infrastructure construction, and industrial construction. However, building construction still lagged behind the other two sectors in the uptake of GPS. 16% and 9% of the building sector and the infrastructure sector respectively did not use GPS in their construction projects. All respondents from the industrial sector acknowledged they used GPS in their construction projects at different degrees.

#### 3.1.2. Radio Frequency Identification (RFID) Technology

RFID technology was far less prevalent in the construction industry compared to GPS. 75% of respondents in the building sector didn’t use RFID in their projects at all. However, about half of the respondents from the infrastructure and industrial sectors were using RFID at different levels.

#### 3.1.3. Ultra-Wideband (UWB) Technology

UWB technology had the lowest implementation level among all eight sensing technologies. 82%, 77%, and 58% of respondents from the building sector, infrastructure sector, and industrial construction were not using UWB at all, respectively.

#### 3.1.4. Fiber Optic Sensing (FOS) Technology

FOS technology is more prevalent in industrial construction than in building or infrastructure construction from the descriptive analysis of the current status. 72% and 69% of projects in the building and infrastructure sectors respectively were not involved in any form of FOS technology implementation. However, 70% of industrial construction projects employed FOS technology at least at some level.

#### 3.1.5. Pressure Sensing Technology

Pressure sensing technology was more prevalent in industrial construction compared to building and infrastructure construction. 11% of industrial sector respondents acknowledged they used pressure sensors in their construction projects on a daily basis. However, only 3% of respondents from the infrastructure sector used pressure sensing technology and none of the building construction respondents used pressure sensors.

#### 3.1.6. Temperature Sensing Technology

Temperature sensing technology had a high level of implementation in all three construction sectors to achieve environmental monitoring. More than half of the respondents in each sector were using it in their construction projects at some level.

#### 3.1.7. Visual Sensing Technology

Visual sensing technology is one of the most popular sensing technologies in construction. 75% of the building construction respondents acknowledged they used visual sensing technologies at some level. The rates rose to 83% and 86% respectively in infrastructure and industrial construction projects.

#### 3.1.8. Three-Dimensional (3D) Scanning Technology

The current status of 3D scanning technology was investigated separately from other visual sensing technologies because of increasing attention from the construction industry. 3D scanning technology was implemented more in industrial construction projects than in building or infrastructure. 22% of industrial construction projects used 3D scanners on a daily basis while the data was just 3% in building and infrastructure construction. Only 14% of respondents from industrial construction didn’t use 3D scanners, while the ratio was 44% and 49% from building and infrastructure construction respectively.

The current status of various sensing technologies in the construction industry containing building, infrastructure, and industrial sectors are concluded in Table 2, Table 3 and Table 4, respectively. A cross-sector comparison on the current status of implementation reveals industrial construction leads employment of sensing technologies, whereas building construction is far behind. The analysis also shows that even popular technologies such as GPS and visual sensing technology are not adopted by many building and infrastructure construction companies. The adoption rate of other sensing technologies such as RFID or FOS technology is even lower than GPS and visual sensing technology.

### 3.2. Influence Factors on Sensing Technologies Adoption in Construction

#### 3.2.1. Partial Least Square Structural Equation Modelling (PLS-SEM)

**Figure 3:**
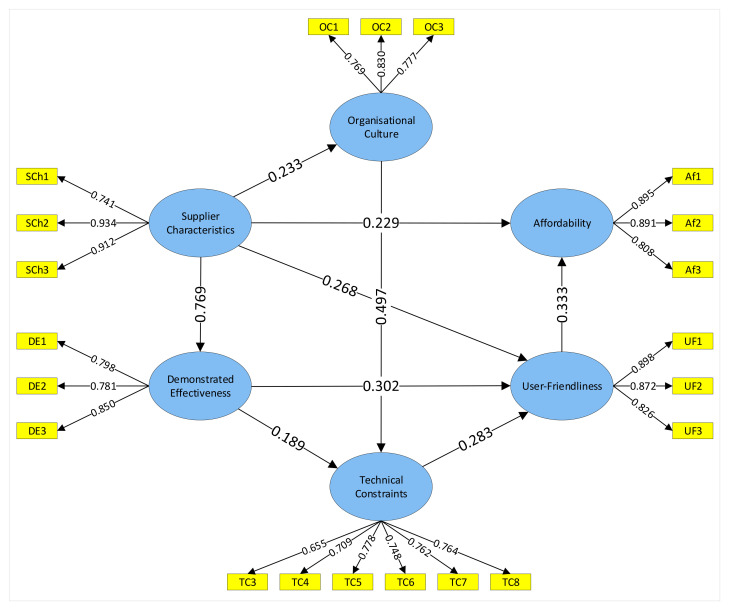
PLS model with factor loadings and path coefficients.

The main goal of the quantitative data collection was to find out the factors significantly influencing sensing technologies adoption from Table 1. The importance of factors categorized into six groupings was rated by construction professionals in an online survey. Research hypotheses on the significant influences between factor groupings were represented as paths in the conceptual framework (Table 5). The structural model was initially defined comprising of nine paths describing the relationships between the six constructs. PLS-SEM was employed to examine the significance of the 24 factors in the framework and hypothesized interrelationships between factor groupings [71]. Then the measurement model was specified to represent how the constructs are measured through the 24 independent variables. All of the six constructs were measured reflectively because the independent variables represent the manifestation of the relevant construct [72]. There are various software programs for running PLS-SEM algorithms however SmartPLS 3 is reported to be the most comprehensive software for the purpose of this research [73]. Data from the 82 responses was assigned to the PLS model and then PLS algorithm calculations followed by bootstrapping techniques were used to estimate the loadings for the measurement model and the structural model. After a few trials of model estimation, three indicators (TC1, TC2, and TC9) were removed from the model since they were not significant or harmful in the PLS model. Lastly, the final model with the factor loadings and path coefficients is presented in Figure 3.

#### 3.2.2. Rank and Discussion of Influence Factors

The rank of identified factors according to their mean scores helps validly interpret perceptions from construction stakeholders on the importance of such factors. A one-sample t-test concluded that the significance of every factor was higher than the test value of 2 (factor being slightly important). Therefore, the value of “Significance” was set as zero to represent that all factors significantly influenced sensing technologies adoption. The mean scores of factors used in the structural equation model ranged from 2.55 to 4.34 (Table 6). Eight factors had a mean score higher than 4 indicating the factors were highly important. The three top-ranked factors were “simplicity of use”, “proof of effectiveness in similar projects”, and “easiness of handling data”.

“Simplicity of use” indicated the high importance to make sure that the new sensing technology didn’t require much effort after implementation and in operation. “Simplicity of use” has been reported when scanner technology is accepted in construction and BIM adopted in medium-size companies [74]. Besides, Usman et al. [49] reported that “operational difficulties” negatively affect the information and communication technology innovation in construction. “Proof of effectiveness in similar projects” was ranked second, showing that the construction professionals were interested in finding successful examples in other construction projects. The successful implementation of new sensing technology in one construction project can positively affect its adoption in other projects and by other construction companies. Lastly, “easiness of handling data” was ranked as the third most important factor, suggesting that management and processing of data is a major concern.

## 4. Qualitative Data Collection and Analysis

The qualitative approach was dedicated to complementing the factors affecting sensing technology adoption and the concerns considered in the implementation phase. Interviews were reported to be the most common method of data collection in qualitative research [75]. The semi-structured interview could provide value-adding information from the perceptions of construction professionals well experienced in sensing technologies. The value-adding information covered factors preventing and motivating sensing technology adoption in construction. Then, factors influencing new sensing technologies adoption were explored, along with desired betterment to a straightforward adoption and efficient implementation.

### 4.1. Design of the Interview Questionnaire and Data Analysis

**Figure 4:**
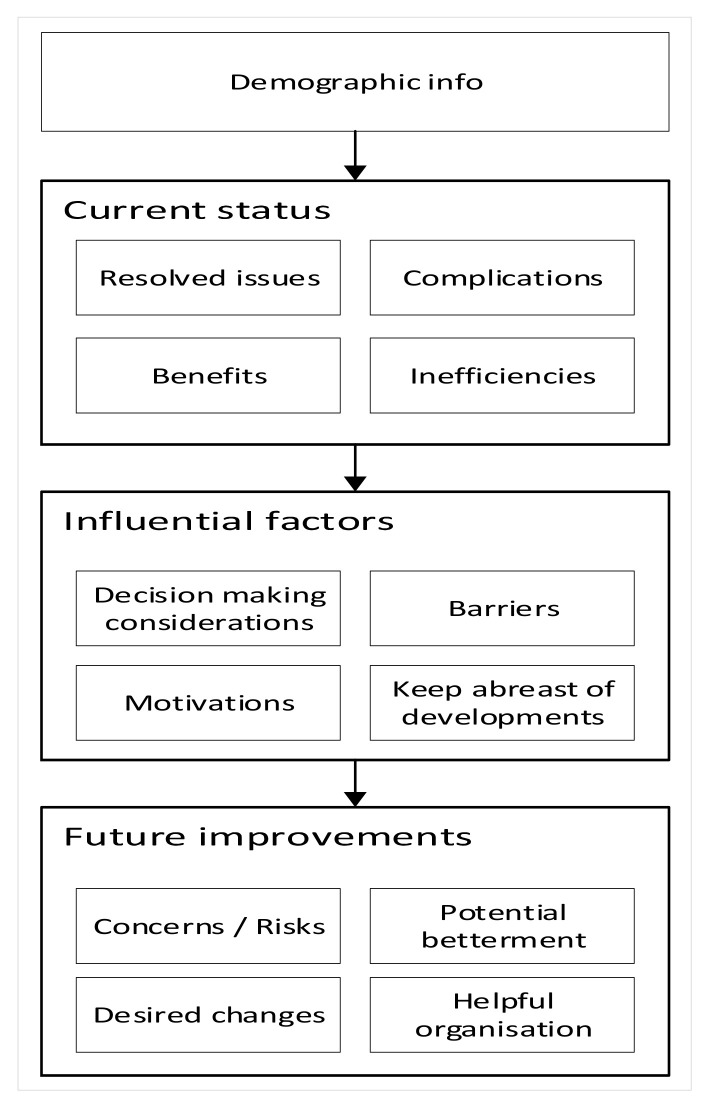
Structure of the questionnaire.

Data collection is regarded as a series of interrelated activities designed to acquire information to answer a research question [76]. An interview questionnaire was designed to collect information on the applications, implementations, benefits, and possible issues of new sensing technologies. The overall structure of the interview questionnaire is illustrated in Figure 4. Interviewees were the construction professionals, especially construction managers and decision makers who were well experienced in sensing technologies. Qualitative data analysis right after each interview showed a noticeable decrease in the number of new nodes being created after the fifteenth interview. Since the data saturation was met by seventeen interviews, no further interview recruitment was pursued.

**Figure 5:**
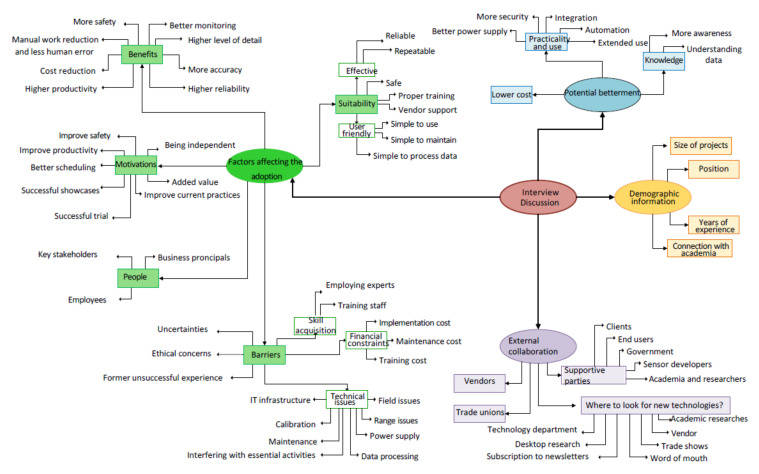
Code of interview transcriptions.

NVivo Pro, which is developed to manage coding procedures for data classification and management, is highly regarded for qualitative data analysis [77]. There were distinct levels of coding of the data in this research. The first level of coding was in close relation to the original text and was intended to guide later and higher coding levels. Recoding continued until a satisfactory level of data classification was acquired. Subsequent levels of coding and recoding finally provided a sophisticated level of coding that comprehensively covered detailed viewpoints of interviewees. As relationships were discovered, nodes were created, and relevant passages assigned to those nodes. Whenever a few nodes related to a specific concept or area of technology adoption they were categorised into a theme. Eventually, four overarching themes became obvious and assisted data interpretation. “Demographic information” provided details on the profile of respondents. “Factors affecting the adoption” was the major focus of the interview discussions and embraced the majority of coding. “Potential betterment” incorporated potential improvements the interviewees were eager to see in regard to the state of sensing technologies in construction. “External collaboration” was basically the collaboration between the construction companies and external parties that might affect the adoption of sensing technologies. More themes are contemplated in each overarching theme. The coding of interview transcriptions undertaken for this research was mapped in Figure 5.

### 4.2. Results and Discussion

The results and interpretation of the qualitative data analysis are presented in this section. The data interpretation involved conceptualizing a larger meaning of data beyond the codes and themes. The discussion analysed the factors affecting the adoption along with related limitations, motivations, and potential betterment towards wider adoption.

#### 4.2.1. Factors Affecting the Adoption and Implementation

Five themes including benefits, barriers, suitability, motivations, and attitude of people were identified from 295 passages about influencing factors. The 295 passages were identified and assigned to 48 nodes through coding the data in NVivo. A few nodes representing a mutual concept were assigned to the parent node.

##### Benefits

**Figure 6:**
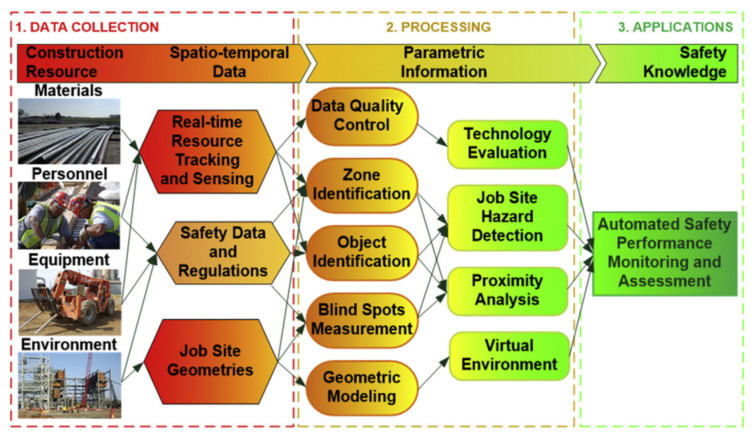
Data collection, information processing, and application framework of RTLS [52].

Benefits resulting from sensing technologies during the construction phase were extensively discussed during the interviews. For example, Real-Time Locating Systems (RTLS) are based on wireless technologies including Wi-Fi, Bluetooth, GPS, Radio Frequency Identification (RFID), etc. The data collection, information processing, and application framework of RTLS are presented in Figure 6. RTLS present great benefits in construction management on the process, safety, and on-site resource through locating and tracking construction materials [78]. Combining with artificial intelligence, the data derived from sensors can be efficiently and effectively analysed to guide the design the construction [13,79,80]. Eight independent nodes of recognized benefits associated with using sensing technologies in construction were identified as follow:

First, a higher level of safety than traditional practice was the primary benefit generated by sensing technology adoption. 31 references were made to the benefit of safety across fifteen interviews. More productive construction processes were the second most noted benefit with 22 references from twelve interviews, particularly on supply chain tracking and material deliveries monitoring. Third, better process monitoring than traditional practices had been noted in twenty references from ten interviews. Fourth, accuracy by sensing technologies adoption had been noted in nine references from seven interviewees. Fifth, manual work reduction resulting in less human error and less rework than traditional practices were another benefit indicated by six interviewees. Sixth, cost reduction was another benefit made in six references from three interviews. Seventh, a higher detail level than traditional practice was concluded by four interviewees. Eighth, higher reliability than traditional practice could be realized via sensing technologies as three interviewees put forward. 

##### Barriers

**Figure 7:**
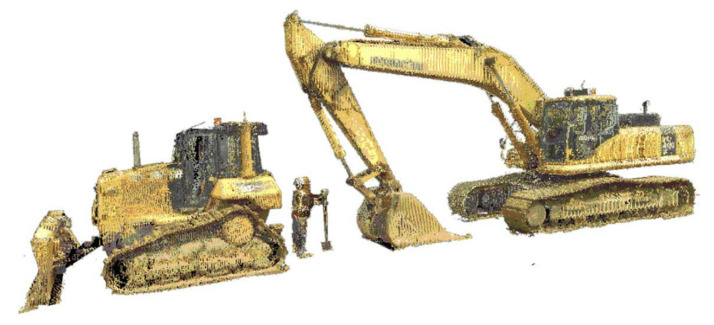
Hazardous situation of worker and equipment measured by a 3D laser scanner [86].

Barriers are those challenges or limitations preventing construction companies from adopting sensing technologies. For example, Laser scanners capture detailed geometries and environmental conditions through laser signals emitted from a rotating laser photon source [28,81]. The hazardous situation of workers and equipment measured using a 3D laser scanner is demonstrated in Figure 7. However, the implementation is restricted by a clear line-of-sight requirement, long data processing time, and high data storage capacity. Sensing technologies are also employed to monitor the mechanical properties of the concrete and steel structures [82,83,84,85]. The theme of barriers to adopting technology was the second most noted concern with 74 references from sixteen interviewees.

First, technical issues were regarded as the most noted barrier with 30 references across eleven interviews. Child nodes of technical issues contained “field issues”, “range issues”, “power supply”, “data processing”, “maintenance”, “calibration”, “Information Technology infrastructure”, and “interference with essential activities”. The second most noted barrier was financial constraints, with 21 references from nine interviews. The parent node “financial constraints” was categorized into three child nodes: “implementation cost”, “training cost” and “maintenance cost”. Third, uncertainties about new sensing technology were put forward with nine references in six interviewees. Fourth, four of the interviewees made six references to concerns about individual privacy and ownership of data when people were involved in data collection. Fifth, skill acquisition was defined as another parent node, with child nodes “training staff” (raised by three interviewees) and “employing experts”, mentioned by two interviewees. Sixth, two interviewees mentioned how their former unsuccessful experience with innovative technologies caused some resistance to accepting new sensing technologies.

##### Suitability of the Technology

**Figure 8:**
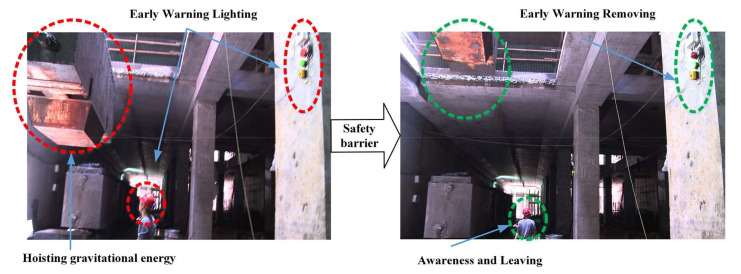
Hazard energy monitoring and safety barrier response in the tunnel shaft area [87].

Suitability of the technology with 59 references was the third most noted concern regarding sensing technology adoption in construction. For instance, RFID technology can secure construction safety when the gate crane driver can’t observe the workers at the bottom of the tunnel shaft [87]. The RFID tags on helmets and RFID readers around this area will generate safety warnings for the potential hazard during vertical transportation (Figure 8). Then the nearby workers would be informed to leave this hoisting area. Ten different nodes were defined within this theme including two parent nodes and three independent nodes.

The first parent node was the effective improvement in construction performance, which was an attribute making a sensing technology suitable for the job. This node attracted 26 references from fourteen interviews, inclusive of two child nodes: “reliable” (ten references from eight interviewees), and “repeatable” (only one reference). The second parent node was the user-friendly technology including three child nodes such as “simple to use”, “simple to maintain”, and “simple to process data”. “Simple to use” attracted eight references from five interviews. “Simple to maintain” resulted from two references mentioned by two interviewees. “Simple to process data” had one reference.

The independent nodes were “being safe” for the construction site (fifteen references from eight interviewees), “proper training” (six references from five interviewees), and “vendor support” (one reference). Besides, safety was mostly mentioned by interviewees from the oil and gas industry as hydrocarbon processing facilities demand intrinsically safe equipment.

##### Motivations

**Figure 9:**
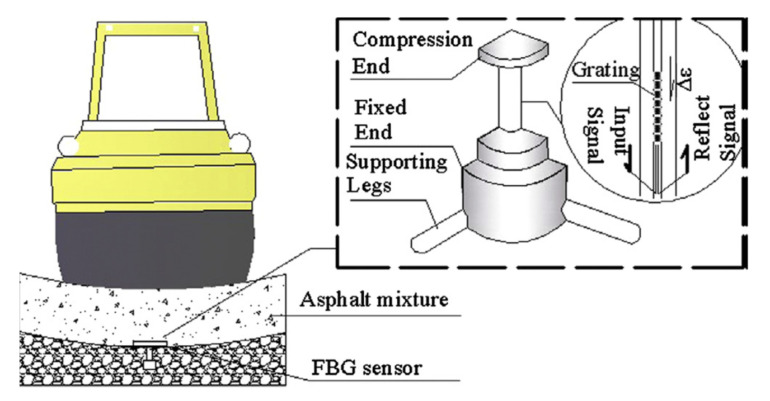
FBG sensor embedded in asphalt pavement [94].

Motivations persuade construction companies to adopt new sensing technologies to improve construction performance on safety, productivity, and quality. Fibre Bragg Grating technology (FBG) realizes real-time and convenient quality control for asphalt mixture compaction operation during lab experiments (Figure 9). FGB technology present great benefits on monitoring the quality of concrete [88,89,90]. Sensing technologies also invite digital technologies and artificial intelligence into the construction industry to predict the mechanical properties under static and flexural fatigue loading [91,92,93]. Forty references related to motivations were detected in fourteen interviews and assigned to eight nodes.

First, construction productivity improvement has been recognized as the most noted motivation initiating sensing technology adoption. The concept of improving productivity as a result of using sensing technologies was mentioned by ten interviewees over seventeen occasions. Safety improvement is the second most noted motivation identified with seven references from six interviews. Third, better schedule monitoring than traditional practice was a motivation to adopt suitable sensing technologies as mentioned by four interviewees. Fourth, added value to the project was mentioned as a motivation by three interviewees. Fifth, a trial period to fit new sensing technology can be a motivation as mentioned by three interviewees. Sixth, independence from third parties was identified as a motivation by two interviewees. Seventh, successful showcases were recognized by two interviewees. Eighth, improvement on current construction practices had been twice mentioned by one interviewee.

##### Attitude of People

**Figure 10:**
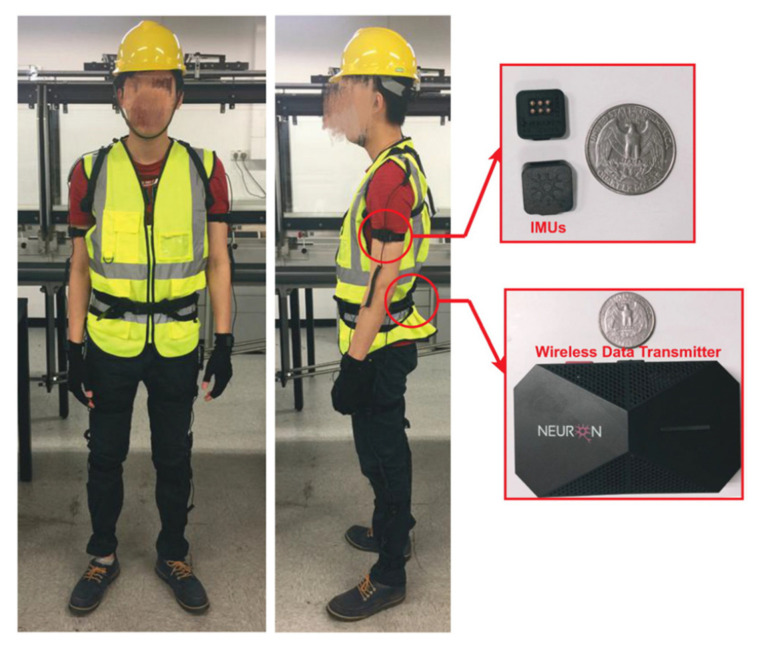
IMU-based wearable motion capture system (Perception Neuron) [97].

The attitude of people towards sensing technologies has been recognized as an important aspect affecting sensing technologies adoption in construction. Some level of resistance might be expected from key stakeholders and employees especially in construction management since these technologies are relatively new. For instance, wearable sensing technologies attached to personal protective equipment realize safety risks detection and health monitoring. Inertial Measurement Units (IMUs) are the most common motion sensors in personal protective equipment to detect awkward postures [95], gait abnormalities [96], and fall-risk assessments [97] (Figure 10). Workers show great willingness to use wearable sensors if data is only collected during working hours [98]. Attitude involved “business principles” (eight references), and a parent node “resistance to change” between “key stakeholders” (nine references) and “employees” (four references). Resistance to change was raised by seven interviewees as a major barrier to sensing technology adoption in construction.

#### 4.2.2. Potential Betterment

Potential betterment, as opposed to benefits, is possible changes and future improvements suggested by the interviewees to improve the application of the sensing technology. Three themes including “practicality and use”, “knowledge”, and “lower cost” were concluded from the coding on the interview transcriptions. 43 passages mentioned the improvements required for better adoption or getting the most out of existing sensing technologies in the interview transcriptions. Eight nodes concluded from the 43 passages were defined and sorted into the three themes.

##### Practicality

The practicality of sensing technologies in construction was the major potential betterment identified in the interview transcriptions. Five child nodes were defined in the context of practicality as “extended use”, “automation”, “integration”, “better power supply”, and “more security”.

First, nine references were made to a desire to see wider use of existing sensing technologies than today. For instance, RFID badges for location access were expected to log into construction equipment, and record time, location, and work. Second, the integration of several sensing technologies into one multidisciplinary system capable of addressing issues across various disciplines was identified as an important desired improvement. Integration meant moving towards the goal of having a unified and integrated sensing system capable of supporting multiple disciplines. Third, the need for some level of automation in construction was identified as a potential betterment in the future by three of the interviewees. The construction industry could benefit from automated field data collection, or even autonomous or semi-autonomous equipment modified and adjusted to fit on construction sites. Lastly, the two final child nodes referred to the need for “better power supply” before recharging is needed, and “more security” against hackers and malware.

##### Knowledge

“Knowledge” of the technology referred to the theme of factors regarding potential betterment in the adoption and implementation of sensing technologies. Fifteen passages discussed different knowledge-based factors towards the wider use of sensing technologies in construction. These factors were “more awareness” towards the benefits of sensing technologies and “understanding data” to get the most out of the technology.

Seven interviewees mentioned the need for more awareness of innovative sensing technologies than previous practice in construction. Besides, twelve occasions emphasized the importance of educating people about the existence and benefits of sensing technologies on construction processes improvement. The importance of mindset openly accepting new technologies was brought up as a key component to accommodate a more straightforward sensing technology adoption. Finally, correct understanding and proper employment was the other aspect of knowledge required for easier technology adoption.

##### Lower Cost

Cost reduction especially on technology procurement was recognized as a potential encouragement for construction stakeholders to try new sensing technologies. Six interviewees noted that more construction companies would try innovative sensing technologies when implementation cost was lower. Therefore, the lower cost was a potential betterment to encourage the adoption of sensing technologies.

#### 4.2.3. External Collaboration

A wide range of interview discussions were around collaboration with external parties. Seventy passages were identified from fifteen interviews talking about external collaboration in sensing technology adoption during construction. Four separate themes were defined to categorize the seventy references on external collaboration.

First, external collaboration in new technologies identification. Information sources to keep abreast of the latest sensing technologies were identified as a combination of the following: “word of mouth”, “vendor”, “desktop research”, “subscription to newsletters”, “technology department”, “trade shows” and “academic research”.

Second, supportive parties could influence the wider adoption of sensing technologies. The supportive parties were identified as end-users, government, and ministerial authorities, sensor developers and suppliers, academia and researchers, and clients. The majority of the interviewees believed that sensor end-users (construction companies) can provide the most constructive feedback to suppliers to improve the technology.

Third, the importance of vendor support and the level of communication with vendors were emphasized by a few interviewees. External collaboration with vendors is recognized as an important factor affecting technology adoption by providing technical support during the implementation, operation, and maintenance.

Fourth, external collaboration with trade unions. One interviewee indicated that trade unions might impose some complications to the sensing technology adoption process, since using sensing technologies involved data collection from people.

## 5. Development and Validation of the Framework

The governance framework was envisaged to highlight the benefits from sensing technologies, foresee possible risks, and generally facilitate the adoption decision-making process. Identified factors from quantitative and qualitative data approaches were combined into a unified system by triangulation analysis to develop the governance framework. This framework will be regarded as feasible after being evaluated and validated by construction professionals.

### 5.1. Triangulation Analysis

A multi-method approach or the use of two or more research methods to investigate a single research question is traditionally called triangulation. Triangulation achieves more unbiased inferences by eliminating or reducing the usual disadvantages of single techniques. Triangulation analysis was used to integrate findings from the literature review with the results of both quantitative and qualitative approaches. The triangulation analysis could strengthen the input into the governance framework, which accommodated various streams of factors affecting the adoption.

**Figure 11:**
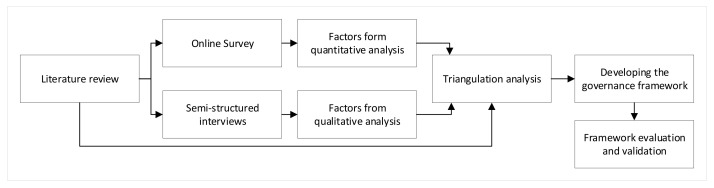
Triangulation method for the governance framework development.

The triangulation method combining quantitative and qualitative results for input into the governance framework was depicted in Figure 11. Significant factors from the quantitative analysis were first considered as the initial input to triangulation. Besides, the remaining factors from the qualitative analysis were entered into the triangulation to reflect more in-depth and detailed information on sensing technology adoption. In other words, factors that were statistically significant in the Partial Least Square model formed the primary input and were consolidated by the factors extracted from the thematic analysis of the interview discussions. The combination of the two approaches was then cross-checked against the literature review and strengthened by any overlooked factor. The categories created from the triangulated results were shown in Table 7.

### 5.2. The Governance Framework

A proposed governance framework would be developed from the triangulation results. To begin with, the core structure of the usual adoption processes comprising of four phases: proposal, evaluation, approval, and implementation. Then, six categories of factors resulting from the triangulation were assigned to relevant phases in the core structure. Next, factors from the survey analysis and qualitative analysis were assigned to relevant categories. Lastly, the initial governance framework was conducted.

Proposal, evaluation, approval, and implementation were identified as the core structure of the framework in interview discussions about usual adoption processes. The sensing technology adoption process began with a proposal on the applicability and suitability of new sensing technology. The proposal needed to be evaluated and approved by decision-makers before implementation. Benefits and motivations towards new sensing technology implementation will promote and expedite the adoption during the evaluation of the proposal. On the other hand, barriers will impede and challenge new sensing technology adoption.

**Figure 12:**
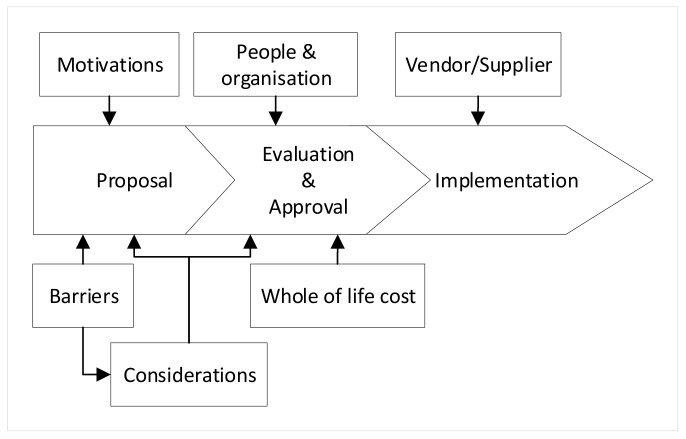
The core structure and categories of factors in the proposed governance framework.

Six categories of factors resulting from the triangulation were assigned to relevant phases in the core structure to develop the framework. The six categories of factors in the proposed governance framework were “motivations”, “barriers”, “considerations”, “people and organization”, “whole of life cost” and “vendor/supplier” (Figure 12). Motivations and barriers are usually well thought out in the proposal phase while the considerations are usually involved during both proposal and evaluation. Barriers constitute a major part of the consideration, so they still affect the evaluation and approval phase. Factors related to people as well as the cost of the technology adoption are mostly considered during the evaluation phase. Suppliers or vendors can assist with a facile adoption when the new sensing technology goes through the implementation phase.

**Figure 13:**
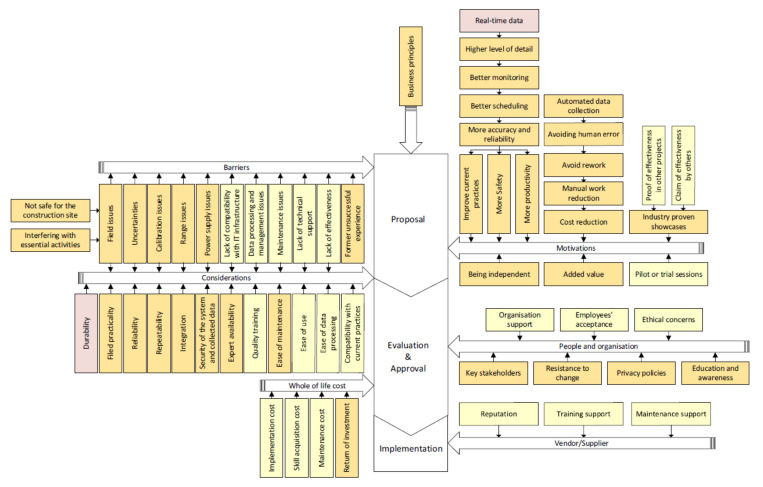
The proposed governance framework.

The last step to develop the proposed governance framework was to assign identified factors affecting the adoption to relevant categories. The 21 factors extracted from the survey analysis are presented in yellow boxes. Besides, the 37 factors extracted from the qualitative analysis are shown in orange except for “vendor/supplier”. The proposed governance framework was conducted resulting from the triangulation analysis was presented in Figure 13.

### 5.3. Evaluation and Validation of the Framework

The proposed governance framework is partially covered and supported by the Automated Data Collection implementation frameworks [70]. Meanwhile, this governance framework is more comprehensive that embracing both positive and negative factors affecting the adoption. The proposed governance framework was presented to construction professionals for evaluation and validation. The evaluation part was designed to identify possible missing factors in any categories and improve the proposed governance framework. The validation part was designed to investigate the comprehensiveness and applicability of the framework to assist construction stakeholders with easy adoption.

#### 5.3.1. Improvement on the Proposed Governance Framework

**Figure 14:**
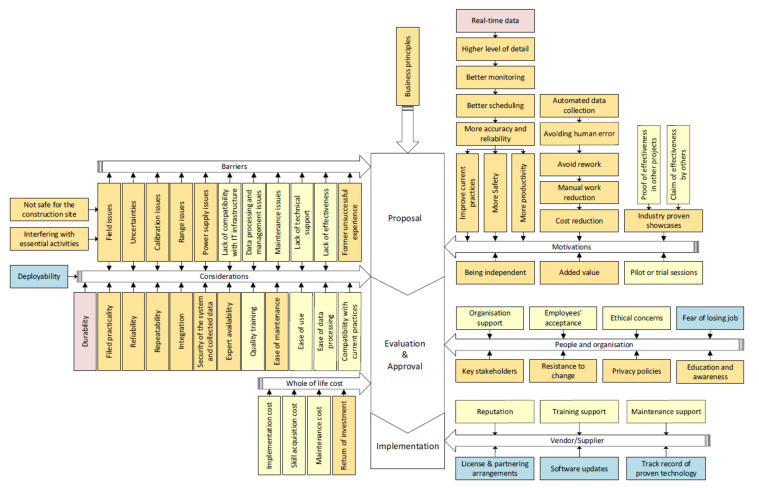
The governance framework.

An online survey was conducted to find out missing factors in any of the categories of the proposed governance framework (Table 8). Ten construction professionals completed the online survey, providing feedback for improving the proposed governance framework. Five missing factors were found according to feedback received from respondents. “Fear of losing jobs” is a barrier related to people. “Deployability” of the technology is regarded as a consideration in the adoption process. “Licence and partnership arrangements”, “software updates” and “track records of proven technologies” are related to vendor or supplier. The five missing factors were added to the proposed governance framework as shown in blue to improve the proposed governance framework (Figure 14).

#### 5.3.2. Validation of the Governance Framework

Validation of the governance framework was conducted to fulfill three criteria of completeness, clarity, and helpfulness in the governance framework. Visser et al. [99] suggested the ontology evaluation to investigate epistemological adequacy (clarity, relevance, and completeness), operationality, and reusability. Validation of the proposed governance framework was done through an online survey similar to the evaluation part. The respondents were asked to rate the completeness, clarity, and helpfulness of the proposed governance framework after evaluating the proposed governance framework. Added factors after the improvement of the framework had only improved the comprehensiveness of the framework but hadn’t jeopardized the validity. Therefore, the improved framework is valid in the first place, as long as the proposed governance framework (which lacks five factors) is valid.

Seven questions used for validating the governance framework were presented in Table 9. Mean values equal to and under 3 are not acceptable while mean values around 4 and above are considered satisfactory. All mean score was equal to or higher than 3.9 indicating the respondents positively supported the completeness, clarity, and helpfulness of the governance framework. The governance framework was acknowledged to be valid on completeness, clarity, and helpfulness with all three criteria for the validation satisfied.

### 5.4. Supplementary Frameworks

The supplementary frameworks focused on two specific aspects including motivations towards new sensing technology adoption and the suitability of new sensing technology to purpose. Besides, respondents mentioned that specific frameworks might address general concerns such as “Does it save money or add value?” and “Can someone prove that it works?”.

#### 5.4.1. Motivation Framework

**Figure 15:**
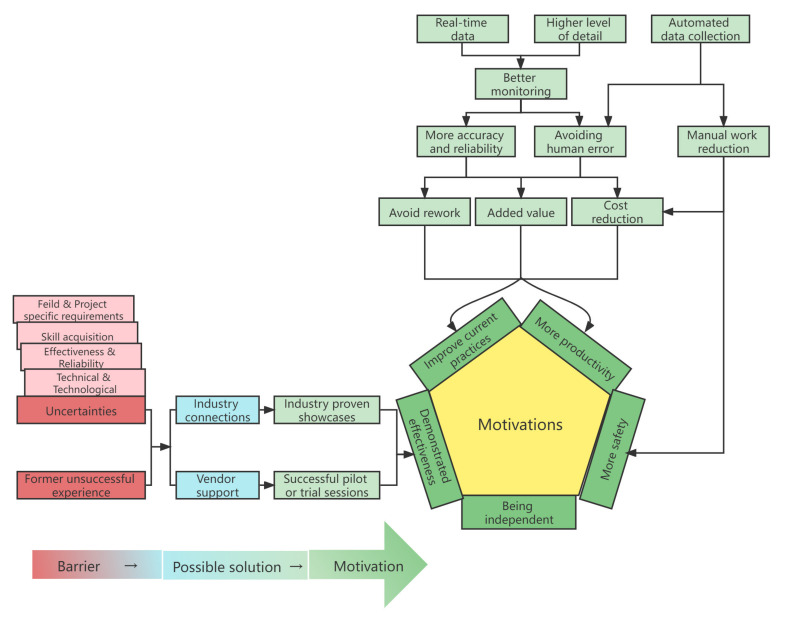
Motivating framework.

The motivation framework has been developed as a secondary outcome of triangulation using the same factors from the governance framework. Five core motivations in the motivation framework accommodated major motivations towards sensing technologies adoption and transformation of barriers into motivations (Figure 15). The relationship between the benefits and motivations of sensing technology adoption was specifically demonstrated in the motivation framework. For example, benefits combined “real-time” data and “higher level of detail” could lead to “avoid rework”, “added value” and “cost reduction” through “better monitoring”. Besides, a possible scenario to transform barriers into motivations was also represented. The “industry connections” or “vendor support” could convert adoption barriers including “former unsuccessful experience” and various “uncertainties” into motivations.

#### 5.4.2. Appraisal Framework

**Figure 16:**
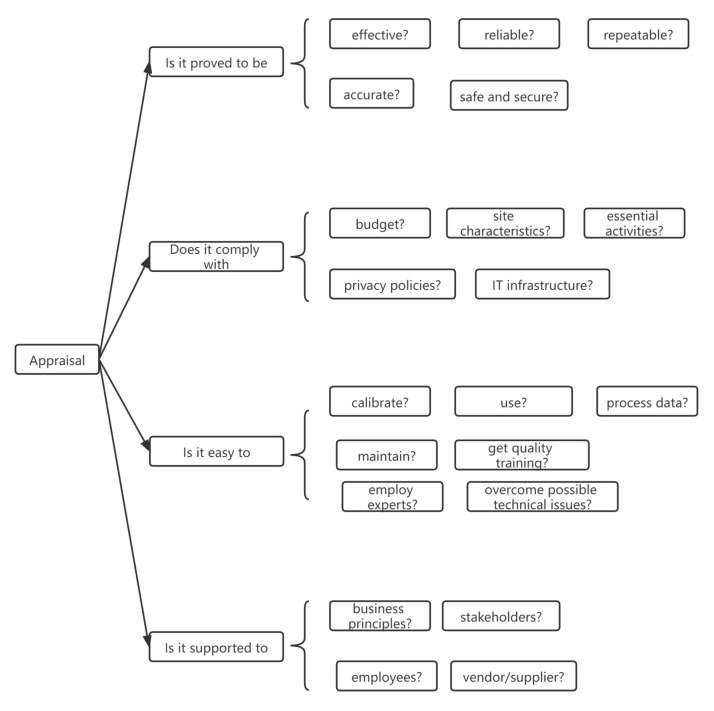
Appraisal framework.

The second supplementary framework was an appraisal framework assessing substantial considerations on the suitability of new sensing technology to the intended purpose. The appraisal framework consisted of four streams of questions to be asked during the proposal and evaluation of new sensing technology (Figure 16). This framework could identify major concerns in dealing with uncertainties to minimize possible risks in introducing a new sensing technology into construction activities. Confidence in the fitness of the proposed sensing technology will increase while the future risks associated with the adoption will reduce.

## 6. Conclusions

In this study, the focus was on those sensing technologies reported to be effective in improving construction safety, productivity, or quality. Eight popular sensing technologies were selected including GPS, RFID, UWB, FOS, pressure sensing, temperature sensing, visual sensing, and 3D scanning technology. The study confirms findings in the literature that the sensing technology adoption in construction projects is slow, despite their huge potential to improve construction performance. An analysis was conducted on the extent to these technologies used in the whole construction industry containing building, infrastructure, and industrial sectors. The research finds that the implementation status of the selected sensing technologies is way behind their capabilities to improve construction performance. A cross-sector comparison on the current status of implementation reveals industrial construction leads employment of sensing technologies, whereas building construction is far behind. The analysis also shows that even popular technologies such as GPS and visual sensing technology are not adopted by many building and infrastructure construction companies. The adoption rate of other sensing technologies such as RFID or FOS technology is even lower than GPS and visual sensing technology.

Awareness of the advantages resulting from the sensing technology implementation is crucial to counter the resistance from the construction industry and promote innovative sensing technologies. The initial step towards these purposes is to develop a governance framework, which is the ultimate aim of this research. The general sensing technologies adoption was the focus of this research rather than specific types of sensors. The framework consists of a core structure depicting the sensing technology adoption process, beginning with a proposal for new sensing technology, followed by evaluation and approval. The implementation phase occurs only when the proposed sensing technology is approved. Various factors are associated with each phase of sensing technology adoption in the governance framework. Barriers and motivations work in opposite directions during the proposal, while considerations lead to minimizing any risk in introducing new devices into the existing systems. The proposal progresses to detailed evaluation and approval when the motivations, barriers, and considerations conclude a new sensing technology is suitable for an intended purpose. Considerations for the suitability, whole of life costs, and factors related to people go to assessment during the evaluation phase. The governance framework can facilitate decision-making on the suitability of particular sensing technology to fit a specific purpose in construction.

Future research could focus on addressing the ethical concerns behind the sensing technologies implementation. For example, trade unions react strongly to personal data collection from employees. A study should cover the points of stakeholders inside and outside of the construction industry. In addition, a case study of the governance framework to any sensing technology in construction could be conducted. As a result, the customized framework extracted from the governance framework will accommodate specific requirements associated with the particular sensor.

## Figures and Tables

**Table 1:** Influence factors on sensing technologies adoption in construction.

Grouping	Indicators	Factors	Reference
**Affordability**	**Af1**	aImplementation cost	[39,40]
	**Af2**	Maintenance cost	[41]
	**Af3**	Skill acquisition cost	[42]
**Demonstrated Effectiveness**	**DE1**	Effectiveness proof from other industry parties	[43]
	**DE2**	Effectiveness proof on trial sessions	[44]
	**DE3**	Effectiveness proof in similar projects	[45]
**Organisational Culture**	**OC1**	Organizational support and approval	[46]
	**OC2**	Acceptance between employees	[47,48]
	**OC3**	Ethical concerns and privacy of employees	[49]
**Supplier Characteristics**	**SCh1**	Reputation of the supplier	[32]
	**SCh2**	Quality training support from the supplier	[50]
	**SCh3**	Quality support from the supplier during maintenance	[51]
**Technical Constraints**	**TC1**	Safety issues	[52]
	**TC2**	Accuracy issues	[53,54]
	**TC3**	Effectiveness issues	[55,56]
	**TC4**	Not adaptable with IT infrastructure	[57]
	**TC5**	High-quality technical support	[58,59,60]
	**TC6**	Hard to too much collected data management	[61]
	**TC7**	High-quality training	[62]
	**TC8**	Hard on maintenance	[63]
	**TC9**	Power supply issues	[64]
**User-friendliness**	**UF1**	Easiness of handling data	[33,65]
	**UF2**	Simplicity of use	[66]
	**UF3**	Compatibility with current systems	[67]

**Table 2:** Current status of various sensing technologies in building construction.

Status	GPS	RFID	UWB	FOS	Pressure Sensing	Temperature Sensing	Visual Sensing	3D Canning
**Daily basis**	20%	7%	0	3%	0	13%	25%	3%
**Frequently**	22%	7%	5%	6%	3%	10%	20%	13%
**Occasionally**	17%	5%	3%	4%	14%	13%	15%	18%
**Very Rare**	25%	6%	10%	15%	17%	15%	15%	22%
**Not at all**	16%	75%	82%	72%	66%	49%	25%	44%

**Table 3:** Current status of various sensing technologies in infrastructure construction.

Status	GPS	RFID	UWB	FOS	Pressure Sensing	Temperature Sensing	Visual Sensing	3D Canning
**Daily basis**	38%	6%	0	4%	3%	15%	19%	3%
**Frequently**	31%	26%	3%	10%	12%	16%	31%	16%
**Occasionally**	13%	9%	7%	7%	12%	21%	15%	19%
**Very Rare**	9%	12%	13%	10%	4%	7%	18%	13%
**Not at all**	9%	47%	77%	69%	69%	41%	17%	49%

**Table 4:** Current status of various sensing technologies in industrial construction.

Status	GPS	RFID	UWB	FOS	Pressure Sensing	Temperature Sensing	Visual Sensing	3D Canning
**Daily basis**	31%	6%	3%	11%	11%	33%	50%	22%
**Frequently**	47%	19%	11%	22%	28%	25%	28%	22%
**Occasionally**	11%	14%	11%	22%	13%	8%	5%	22%
**Very Rare**	11%	11%	17%	15%	20%	6%	3%	20%
**Not at all**	0	50%	58%	30%	28%	28%	14%	14%

**Table 5:** Hypotheses on the significant influences between factor groupings.

Hypotheses	
**1**	Supplier characteristics positively affect organizational culture
**2**	Supplier characteristics positively affect affordability
**3**	Supplier characteristics positively affect user friendliness
**4**	Supplier characteristics positively affect demonstrated effectiveness
**5**	Demonstrated effectiveness positively affects user friendliness
**6**	Demonstrated effectiveness positively affect technical constraints
**7**	Organizational culture positively affects technical constraints
**8**	Technical constraints positively affect user friendliness
**9**	User friendliness positively affects affordability

**Table 6:** Rank of the factors.

Grouping	Indicators	Mean	Significance	Rank
**Affordability**	**Af1**	3.99	0.000	8
	**Af2**	4.09	0.000	6
	**Af3**	3.63	0.000	12
**Demonstrated Effectiveness**	**DE1**	3.83	0.000	10
	**DE2**	4.18	0.000	4
	**DE3**	4.29	0.000	2
**Organisational Culture**	**OC1**	3.65	0.000	11
	**OC2**	3.18	0.000	15
	**OC3**	2.55	0.000	20
**Supplier Characteristics**	**SCh1**	3.95	0.000	9
	**SCh2**	4.06	0.000	7
	**SCh3**	4.13	0.000	5
**Technical Constraints**	**TC3**	3.23	0.000	14
	**TC4**	3.01	0.000	17
	**TC5**	2.68	0.000	18
	**TC6**	3.26	0.000	13
	**TC7**	2.67	0.000	19
	**TC8**	3.06	0.000	16
**User-friendliness**	**UF1**	4.24	0.000	3
	**UF2**	4.34	0.000	1
	**UF3**	4.13	0.000	5

**Table 7:** Categories for triangulated factors.

Category	Factors	Category	Factors
**Benefits** **and** **motivations**	Real-time data	**Considerations**	Durability
	More safety		Field practicality
	More productivity		Reliability
	Improve current practices		Repeatability
	More accuracy and reliability		Security of the system and collected data
	Better scheduling		Ease of maintenance
	Better monitoring		Expert availability
	Higher level of detail		Integration
	Cost reduction		Ease of data processing
	Manual work reduction		Ease of use
	Avoid rework		Compatibility with current systems
	Avoid human error		Quality training
	Automated data collection	**Whole of life cost**	Implementation cost
	Industry-proven showcases		Maintenance cost
	Added value		Skill acquisition cost
	Being independent		Return of investment
	Proof of effectiveness in other projects	**People and organization**	Organization support
	Claim of effectiveness by others		Employees’ acceptance
	Pilot or trial sessions		Ethical concerns
**Barriers**	Field issues		Key stakeholders
	Range issues		Resistance to change
	Power supply issues		Privacy policies
	Calibration issues		Education and awareness
	Uncertainties	**Vendor/Supplier**	Supplier’s reputation
	Former unsuccessful experience		Supplier’s training support
	Not safe for the construction site		Supplier’s maintenance support
	Interfering with essential activities	**Business principles**	Business principles
	Lack of compatibility with IT infrastructures		
	Data processing and management issues		
	Maintenance issues		
	Lack of effectiveness		
	Lack of technical support		

**Table 8:** Questions for evaluation the proposed governance framework.

Number	Question
Q1	Is there any missing motivation to encourage construction stakeholders to adopt sensing technologies?
Q2	Is there any missing barrier limiting the adoption of sensing technologies in construction?
Q3	Is there any missing consideration for the decision-making process of sensing technology adoption and implementation during construction?
Q4	Is there any missing factor affecting the adoption of sensing technologies in construction which is related to people?
Q5	Is there any missing factor related to the role of vendor or supplier in sensing technology adoption?

**Table 9:** Rank of validation questions and the associated mean.

Criterion	Question	Mean	
		For Question	For Criterion
Completeness	Q1. To what extent do you agree that the framework covers all relevant factors for sensing technology adoption?	4.20	4.25
	Q2. To what extent do you agree that all the factors in the framework are relevant to sensing technology adoption?	4.30	
Clarity	Q3. To what extent do you agree that the terminology used within the framework reflects the intuition of experts?	3.90	3.97
	Q4. To what extent do you agree that every factor within the framework is allocated to a proper stage of sensing technology adoption (proposal, approval, and implementation)?	3.90	
	Q5. To what extent do you agree that the concepts (factors) and their relations (classification) used within the framework are clear and explicit enough?	4.10	
Helpfulness	Q6. To what extent do you agree that the framework is capable of assisting construction stakeholders and decision-makers with wider adoption of sensing technologies in construction?	4.20	4.15
	Q7. To what extent do you agree that the framework is usable and re-usable for the adoption of all types of sensing technologies in construction?	4.10	

## Data Availability

The data presented in this study are openly available.
